# Praja2 suppresses the growth of gastric cancer by ubiquitylation of KSR1 and inhibiting MEK-ERK signal pathways

**DOI:** 10.18632/aging.202356

**Published:** 2021-01-10

**Authors:** Zhiwei Zhao, Lin Zhu, Yanwei Xing, Zhenan Zhang

**Affiliations:** 1 Department of General Surgery, The First Affiliated Hospital of Zhengzhou University, Zhengzhou, Henan Province, China; 2 Laboratory of Functional Materials, Henan University of Technology, Henan Province, China; 3 Department of Colorectal Surgery, The First Affiliated Hospital of Harbin Medical University, Harbin, Heilongjiang Province, China; 4 Department of Surgery, The Infectious Disease Hospital of Heilongjiang Province, Harbin, Heilongjiang Province, China

**Keywords:** gastric cancer, E3 ligase praja2, KSR1, MEK-ERK, tumorigenesis

## Abstract

Gastric cancer (GC) is a common malignant tumor, which has a high incidence and fatality. Therefore, it is important to clarify the molecular mechanism of the occurrence and development for GC and to find more effective treatments and targeted drugs. In this study, we found that the kinase suppressor of Ras1 (KSR1) was increased in GC tissues and cell lines. Silencing of KSR1 inhibited the proliferation, migration and invasion of MKN-45 cells. E3 ligase Praja2 was downregulated in GC tissues and cell lines. In addition, praja2 promoted ubiquitylation of KSR1, but inhibited MEK-ERK signal pathways. Functional analysis indicated overexpression of praja2 inhibited the proliferation, migration and invasion of MKN-45 cells, while MG132 or FGF2 treatment removed the inhibitory effects of praja2 on GC progression. *In vivo* tumorigenesis experiments indicated praja2 inhibited tumor growth via KSR1-MEK-ERK axis. In conclusion, praja2 promoted the ubiquitylation and degradation of KSR1, which disturbed MEK- ERK signaling and inhibited GC progression. Our study might provide a novel target for GC clinical treatment.

## INTRODUCTION

Gastric cancer is the second leading cause of digestive tract malignant tumor in the world [1]. More than 70% of new cases and deaths occur in developing countries [2], of which China accounts for about 42% of new cases of gastric cancer in the world [3]. At present, the pathogenesis of gastric cancer is not clear, and the research on the pathogenesis of gastric cancer is changing with each passing day [4]. Therefore, it is very important for the diagnosis and treatment of gastric cancer to find effective early warning molecules, improve the early diagnosis and find out the molecular mechanism of invasion of gastric cancer.

With the development of science and technology, epigenetic regulation has attracted more and more attention, including phosphorylation, methylation, acetylation and ubiquitination [5]. Moreover, many studies have pointed out that epigenetic changes of some genes are the key link in inducing or inhibiting tumor formation [6, 7]. Among them, ubiquitination plays an important role in regulating the activities of substrate proteins and enzymes [8], while E3 ligase can effectively recognize and ubiquitinate protein substrates and promote ubiquitin-proteasome degradation [9]. For instance, CHIP can mediate the ubiquitination and degradation of misfolded proteins in cells, especially some tumor-related proteins, thus imaging the occurrence and development of tumors [10]. Studies have shown that CHIP can ubiquitinate ErbB2 using u-BOX structure, thereby weakening the mitosis signaling pathway mediated by ErbB2 and ultimately inhibiting the proliferation of breast cancer [11, 12]. Additionally, CHIP inhibits NF-κB activity, angiogenesis, and gastric cancer invasion and distant migration by binding to NF-κB and promoting ubiquitination mediated by the proteasome [13]. Praja2 is a member of RING E3 ligase, which has been reported to be involved in multiple cell signalings in various cancers. Lignitto et al. reported that praja2 could inhibit Hippo pathway via ubiquitinating MOB1, which accelerates the progression of glioblastoma [14]. In addition, praja2 is up-regulated in thyroid cancer, which is related to a poor prognosis of thyroid cancer [15]. Besides, praja2 contributes to MAPK pathway and ERK pathway, JNK pathway, and these pathways are involved in cancer progression [16]. Nevertheless, the function and mechanism of praja2 in gastric cancer are poorly defined.

Mitogen-activated protein kinase (MAPK) signal pathway is involved in regulating a variety of biological processes, including cell proliferation, differentiation, migration, survival and cell metabolism [17]. The MAPK signal pathway is mainly composed of RAF, MEK and ERK. After being stimulated by extracellular factors such as cellular factors, many downstream substrates are phosphorylated to participate in a variety of signal transduction [18]. Ras kinase inhibitor (KSR) is the most classical regulatory protein of the MAPK signal pathway, which can positively regulate the Ras/MAPK signal pathway [19]. Ras/Raf/MAPK signal pathway is a classic tumor-related signal pathway, and KSR plays a key role in this signal pathway, so many studies have begun to explore the biological characteristics of KSR in different tumors. KSR1 is highly expressed in human endometrial carcinoma, and the growth of tumor cells is inhibited after knockout of KSR1 [20]. Continuous injection of phosphorothioate antisense oligodeoxynucleotides ((ODN)) into K-Ras-dependent human PANC-1 pancreatic cancer cells and A549 non-small cell lung adenocarcinoma xenografts in nude mice can slow down tumor growth, indicating that KSR1 is a potential therapeutic target for Ras-dependent malignant tumors [21]. A recent study illuminates that praja2 ubiquitinates KSR1 and inhibits mitogenic signaling [22]. Therefore, we hypothesized that praja2 ubiquitylates KSR1 to modulate GC tumorigenesis.

Herein, we aimed to explore the effect of praja2 in GC and further illuminate the possible underlying mechanisms.

## RESULTS

### Up-regulation of KSR1 in GC tissues and cells

**Figure 1:**
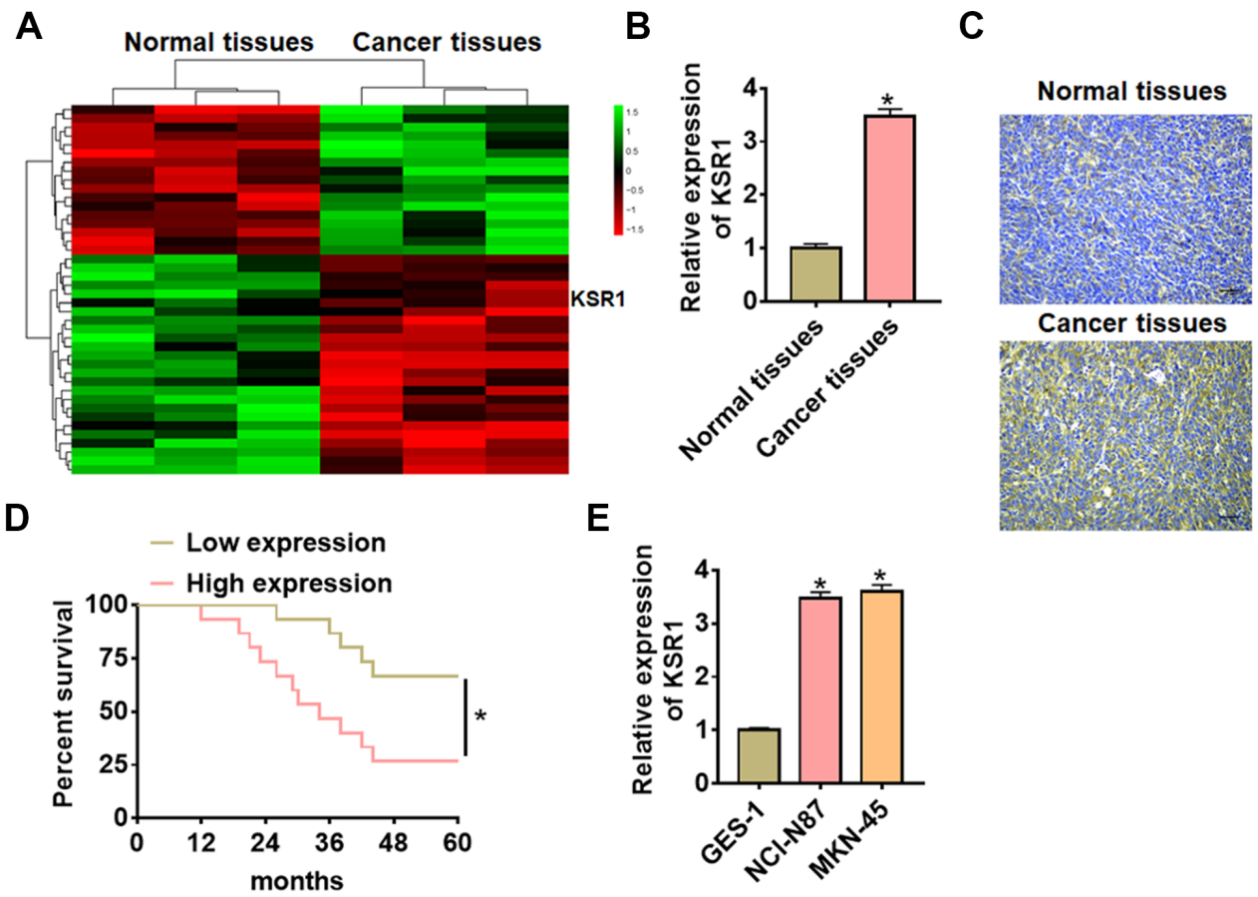
**The expression of KSR1 in GC tissues and cell lines.** (**A**) mRNA expression profiles of normal tissues and cancer tissues in GC. (**B**) The expression of KSR1 in normal and cancer tissues was detected by qRT-PCR. n = 40. (**C**) IHC for KSR1 in normal and cancer tissues of GC. Scale bar, 40 μm. (**D**) According to the median level of KSR1 in Figure 1B, 40 GC patients were divided into low (n = 20) and high expression group (n = 20). Kaplan-Meier curves indicated a 5-year survival rate of GC patients. (**E**) qRT-PCR analysis for KSR1 level in normal gastric mucosal epithelial cell line GES-1 and GC cell lines (NCI-N87, MKN-45). Data are mean ± SD; *P < 0.05.

Firstly, we used bioinformatics to analyze the differentially expressed genes in tumor tissues and adjacent tissues from GC patients (Figure 1A). According to microarray data, KSR1 was significantly increased, suggesting KSR1 may involve in the progression of GC. Then we detected the expression level of KSR1 in 40 paired GC patients. KSR1 was upregulated in GC cancer compared with para-carcinoma tissues (Figure 1B, 1C). According to the median level of KSR1 in Figure 1B, 40 GC patients were divided into low (n = 20) and high expression group (n = 20). Kaplan-Meier curves indicated the 5-year survival rate of GC patients was significantly higher in low expression patients than high expression patients (Figure 1D). In addition, we cultured GC cell lines (NCI-N87, MKN-45) and human normal gastric mucosal epithelial cell line GES-1, and found the level of KSR1 was dramatically higher in GC cell lines than that in the normal gastric mucosal epithelial cell line (Figure 1E). Together, these data indicated that KSR1 might be involved in GC progression.

### Silencing KSR1 inhibited progression of GC cells

**Figure 2:**
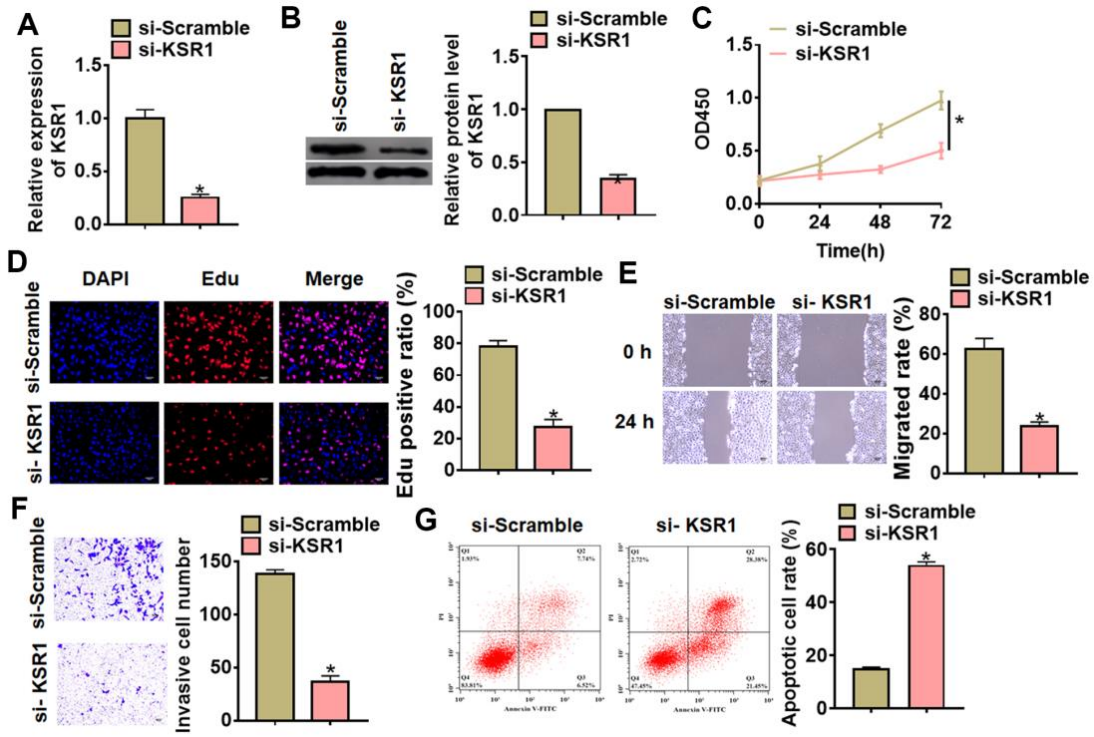
**Silencing of KSR1 inhibited the progression of GC cells.** (**A**) The silencing efficiency of si-KSR1 in MKN-45 cells. (**B**) Western blot was to measure the protein level of KSR1 in MKN-45 cells. (**C**) CCK8 was to determine the viability of MKN-45 cells. (**D**) Edu assay was to detect proliferation of MKN-45 cells. Scale bar, 40 μm. (**E**) Wound healing assay was to evaluate migration of MKN-45 cells. Scale bar, 60 μm. (**F**) Transwell assay was to examine invasion of MKN-45 cells. Scale bar, 60 μm. (**G**) Flow cytometry assay was to determine apoptosis of MKN-45 cells. Data are mean ± SD; *P < 0.05.

To further identify the function of KSR1 in GC progression, we constructed small interfering RNA of KSR1 (si- KSR1). As showed in Figure 2A, 2B, transfection of si-KSR1 significantly suppressed the expression of KSR1 in MKN-45 cells as compared to cells transfected with scrambled siRNA. Then we used CCK8 assay to detect cell viability, and found that si-KSR1 inhibited MKN-45 viability (Figure 2C). Edu analysis was performed to detect cell proliferation, which showed that deletion of KSR1 decreased Edu positive cell numbers (Figure 2D). Then, wound healing suggested that silencing KSR1 decreased the wound healing area, which exhibited a lower migratory ability in si-KSR1 transfected cells (Figure 2E). Transwell assay showed that si-KSR1 reduced the cell invasive ability in MKN-45 cells (Figure 2F). And flow cytometry assay showed an increase of apoptotic cell numbers after silencing KSR1 (Figure 2G) (Supplementary Figures 1, 2).

**Figure 3:**
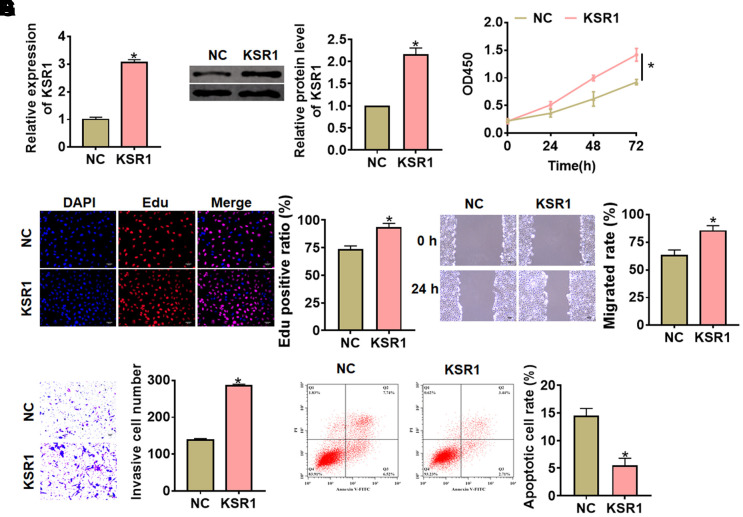
**Overexpression of KSR1 promoted the progression of GC cells.** (**A**, **B**) The transfection efficiency of KSR1 in MKN-45 cells using qRT-PCR and western blot. (**C**) CCK8 for viability of MKN-45 cells. (**D**) Edu assay for proliferation of MKN-45 cells. Scale bar, 40 μm. (**E**) Wound healing assay for migration of MKN-45 cells. Scale bar, 60 μm. (**F**) Transwell assay for invasion of MKN-45 cells. Scale bar, 60 μm. (**G**) Flow cytometry assay for apoptosis of MKN-45 cells. Data are mean ± SD; *P < 0.05.

Next, we constructed KSR1 plasmid to force its expression in MKN-45 cells (Figure 3A, 3B). Followed functional analysis indicated that overexpression of KSR1 promoted cell viability, proliferation, migration and invasion of MKN-45 cells (Figure 3C–3F), but inhibited cell apoptosis (Figure 3G). These results showed that knockdown of KSR1 suppressed GC progression.

### Praja2 ubiquitylated KSR1 and inhibited KSR1-related MEK-EKR signal pathways

**Figure 4:**
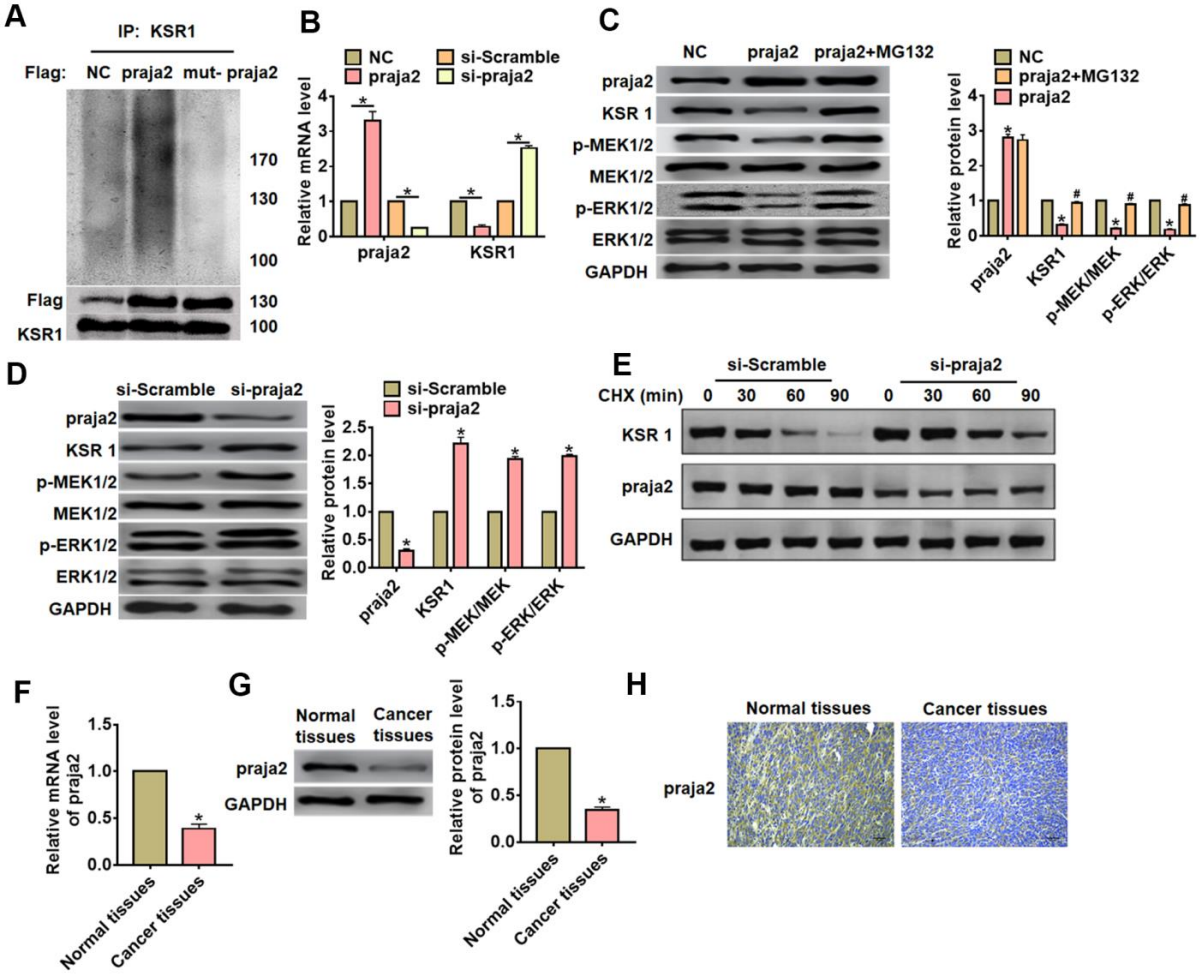
**Praja2 promoted ubiquitylation of KSR1**. (**A**) HA-ubiquitin and Flag- praja2 or Flag-mut-praja2 was transfected into HEK293 cells. IP analysis for the relation between praja2 and KSR1. (**B**) Praja2 or si-praja2 or its NC was transfected into MKN-45 cells, and qRT-PCR analysis for the expression of KSR1. (**C**) Praja2 or NC was transfected into MKN-45 cells with or without the presence of MG132. Western blot was used to detect praja2, KSR1, MEK, p-MEK, ERK and p-ERK protein level. (**D**) si-praja2 or si-NC was transfected into MKN-45 cells, and western blot for the expression of praja2, KSR1, MEK, p-MEK, ERK and p-ERK protein. (**E**) CHX assay was used to determine the role of praja2 on KSR1 stability. (**F**, **G**). The expression of praja2 in normal and cancer tissues was detected by qRT-PCR and western blot. (**H**) IHC for praja2 in normal and cancer tissues of GC. Scale bar, 40 μm.

Previous study has shown that KSR1 can be ubiquitylated by praja2, and praja2 has essential significance in the development of tumor. Thus, we speculated that praja2 might ubiquitylate KSR1 and regulated GC progression. To verify our hypothesis, we transfected HA-ubiquitin and Flag- praja2 or Flag-mut-praja2 into HEK293 cells. The IP results showed that praja2 promoted the ubiquitination of KSR1, while mutant of praja2 inhibited KSR1 ubiquitination (Figure 4A). Then, we transfected praja2 or si-praja2 or its NC into MKN-45 cells, and qRT-PCR analysis showed that praja2 reduced KSR1, while si-praja2 induced KSR1 expression (Figure 4B). As well, overexpression of praja2 inhibited KSR1 protein level and inactivated MEK-ERK pathway, while MG132 (proteasome inhibitor) treatment reversed the effect of praja2 on KSR1 expression and MEK-ERK pathway (Figure 4C). In addition, silencing of praja2 promoted KSR1 level and activated MEK-ERK pathway (Figure 4D). Furthermore, CHX assay showed that si-praja2 promoted the stability of KSR1 (Figure 4E). Next, we evaluated praja2 expression in GC. The results showed that praja2 was decreased in GC tissues than that in normal tissues (Figure 4F–4H).

### Praja2 inhibited GC progression via inhibiting KSR1-MEK-ERK axis

**Figure 5:**
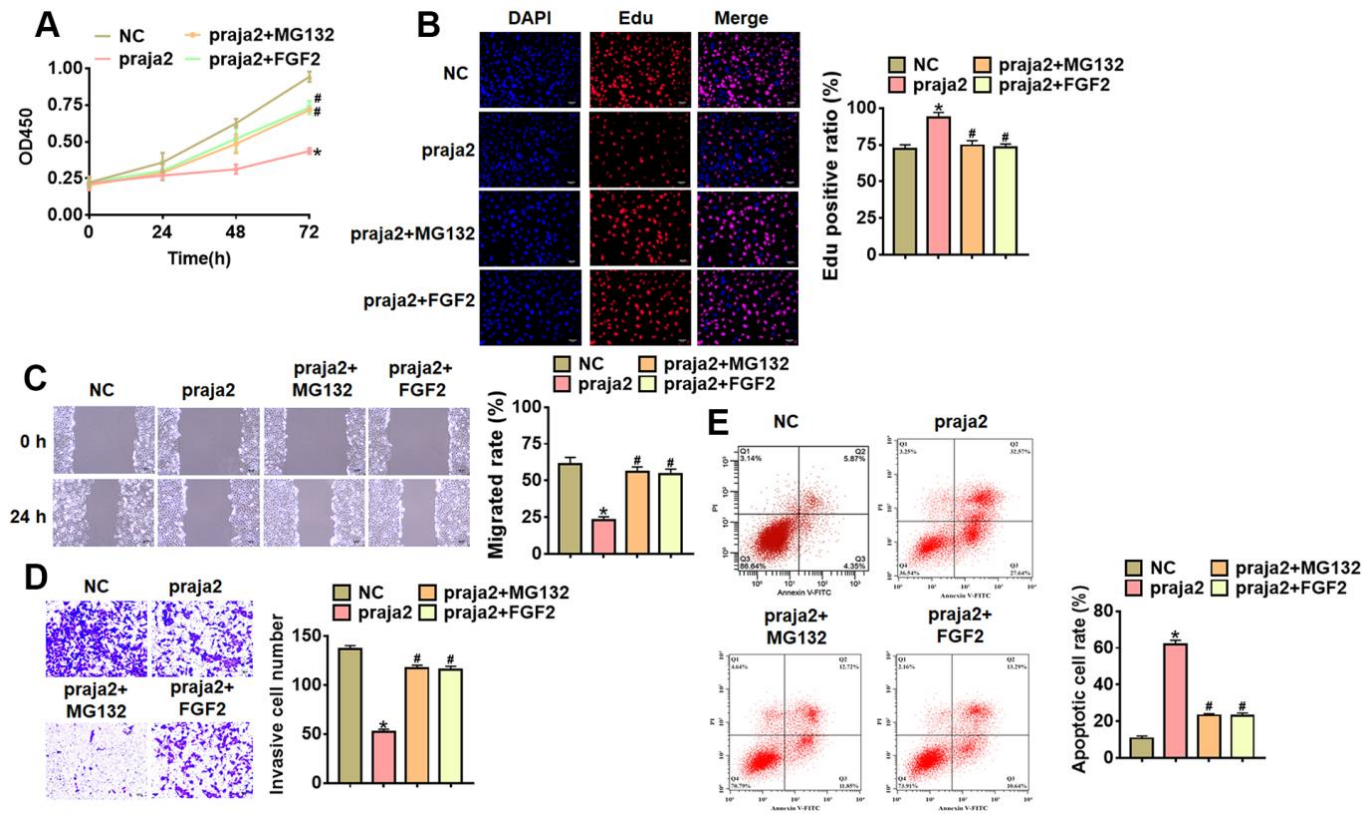
**Praja2 inhibited the progression of GC cells through KSR1-MEK-ERK axis.** Praja2 or NC was transfected into MKN-45 cells, and treated with MG132 or FGF2 (MEK-ERK activator). (**A**) CCK8 for viability of MKN-45 cells. (**B**) Edu assay for proliferation of MKN-45 cells. Scale bar, 40 μm. (**C**) Wound healing assay for migration of MKN-45 cells. Scale bar, 60 μm. (**D**) Transwell assay for invasion of MKN-45 cells. Scale bar, 60 μm. (**E**) Flow cytometry assay for apoptosis of MKN-45 cells. Data are mean ± SD; *P < 0.05.

To further identify the role and mechanism of praja2 in GC progression, we forced praja2 expression in MKN-45 cells (Figure 5A), and treated with MG132 or FGF2 (MEK-ERK activator). Followed functional analysis showed that praja2 inhibited cell viability, proliferation, migration and invasion, and promoted cell apoptosis of MKN-45 cells (Figure 5B–5E), while MG132 or FGF2 treatment remitted the benefit effect of praja2 (Figure 5B–5E). Taken together, praja2 inhibited GC progression via inhibiting KSR1-MEK-ERK axis.

### Praja2 inhibited GC growth

**Figure 6:**
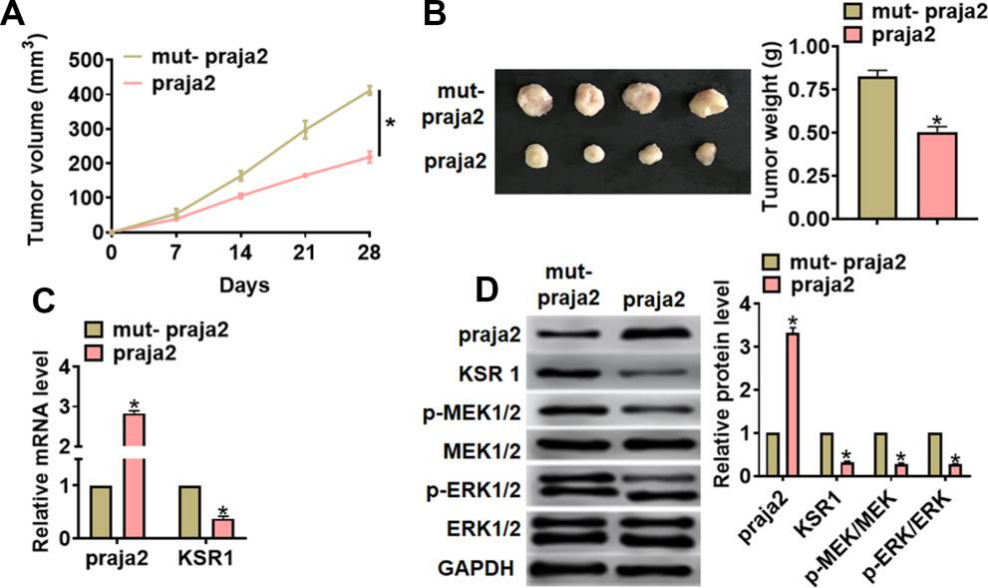
**Praja2 inhibited GC growth *in vivo*.** 30 mice were divided into two group randomly, MKN-45 cells were subcutaneously injected into nude mice. 1 week later, we injected lentivirus packaged praja2 or NC into tumors. (**A**) Tumor volume was measured every 7 days. (**B**) Tumors was isolated after 28 days of MKN-45 cells injection, and photos for representative tumors. (**C**) The mRNA of praja2 and KSR1 in isolated tumors were detected by qRT-PCR. (**D**) Western blot for praja2, KSR1, MEK, p-MEK, ERK and p-ERK protein expression in isolated tumors. Data are mean ± SD; *P < 0.05.

For further explore the function of praja2 in GC, we set up a xenograft nude mice model. 30 mice were divided into two groups randomly, MKN-45 cells were subcutaneously injected into nude mice. 1 week later, we injected lentivirus packaged praja2 or NC into tumors, and we measured tumor volume. The mice with praja2 showed a smaller tumor volume, and tumors grew slower (Figure 6A). The tumors were isolated at 28 days after injection, praja2 significantly decreased tumor weight (Figure 6B). In addition, isolated tumor tissues had a higher praja2 level after injection of lentivirus packaged praja2, and injection of praja2 decreased the mRNA level of KSR1 (Figure 6C). Furthermore, we performed western blot to examine MEK-ERK signaling, and found that injection of praja2 inhabited the activation of MEK-ERK signaling (Figure 6D).

## DISCUSSION

Gastric cancer is one of the most common malignant tumors in China [23]. Among the newly diagnosed cancer cases every year, gastric cancer ranks fourth and ranks second in cancer mortality. Due to unknown etiology, occult onset, lack of specific symptoms and effective early diagnosis, most cases of gastric cancer are diagnosed in the late stage [24]. Coupled with the decrease of sensitivity of gastric cancer cells to radiotherapy and chemotherapy and the increasing drug resistance in recent years, the 5-year survival rate of patients with gastric cancer is less than 20% [25]. Therefore, it is more and more important to find more effective molecular biological markers to improve the early diagnosis rate of gastric cancer. In present study, we revealed that praja2 could inhibit GC tumor growth via KSR1-MEK-ERK axis, which might be a novel target for GC clinical treatment.

As a scaffold protein in Ras/Raf/MAPK signal pathway, KSR1 forms a complex with three kinases Raf/MEK/ERK in MAPK signal pathway on the cell membrane [26]. Under external stimulation, KSR1 increases the phosphorylation of Raf, MEK and ERK, promotes upstream signal transduction and multiple downstream substrates in the cytoplasm and nucleus, and regulates biological processes such as cell proliferation, cell cycle and apoptosis in this way [27, 28]. Recent studies have shown that KSR1 is expressed in many tumor cell lines, such as lung cancer, prostate cancer, breast cancer and so on, and the expression of KSR1 is related to the sensitivity of antineoplastic drugs [29, 30]. And in present study, we performed microarray and found that KSR1 was increased in GC patients, and this result was further confirmed by qRT-PCR. Additionally, high expression of KSR1 indicated a poor prognosis. Tumor cell migration and invasion are important features of advanced malignant tumors, and it is also one of the important factors that determine the prognosis and five-year survival rate of many tumors. In our results, we found that silencing of KSR1 inhibited the proliferation, migration and invasion of MKN-45 cells. On the contrary, overexpression of KSR1 promoted progression of GC.

Recent studies have revealed that KSR1 could be ubiquitinated by E3 ligase praja2 [22]. Ubiquitin-proteasome system plays a key role in regulating intracellular signal pathways by regulating the expression, activity and localization of many endogenous proteins [31]. Ubiquitin can bind to the targeted protein through three enzymes E1, E2 and E3. Among them, E3 is a ubiquitin ligase that cooperates with the transport of ubiquitin to the target protein, which participates in the regulation of tumor growth by mediating the ubiquitin degradation of tumor-related proteins [32]. Studies have shown that the high expression of E3 ubiquitin ligase EDD is positively correlated with recurrence and death in patients with ovarian cancer [33]. CUL4A degrades DBB, through ubiquitin-proteasome pathway to damage the activity of DNA, weakens the ability of DBB to recognize and repair damaged DNA in tumor cells, and finally advances the development of breast cancer [34]. And praja2 also contributed to the progression of tumors and regulated cellular signaling pathway [35, 36]. In present study, we found that praja2 promoted ubiquitylation and degradation of KSR1. And praja2 expression was significantly decreased in GC tissues and cell lines. Furthermore, praja2 suppressed the expression of p-MEK and p-ERK, inhibited MEK-ERK pathway. Because MEK-ERK pathway is a key signal for the biological activity of gastric cancer cell [37], we further explored the role of praja2 in on GC progression. Interestingly, praja2 inhibited the growth and invasion of GC cells, while MG132 or FGF2 treatment removed the inhibitory effects of praja2 on GC progression. This data indicated that KSR1-MEK-ERK axis was downstream of praja2, and praja2 regulated GC development through KSR1-MEK-ERK axis. *In vivo* tumorigenesis experiments indicated praja2 inhibited tumor growth via KSR1-MEK-ERK axis.

## MATERIALS AND METHODS

### Tissue specimen

The surgical specimens of 40 GC patients from our hospital were collected, which were used for follow-up experimental detection. The experiment was permitted by the Ethics Committee of the First Affiliated Hospital of Zhengzhou University and the patients signed informed consent.

### Microarray analysis

LncRNAs in GC tissues were profiled using microarray analysis (Bio-Tech, Shanghai, China). Differentially expressed lncRNAs were identified by Heatmap. Up-regulated or down-regulated mRNAs were selected based on changes ≥ 2 fold threshold and P < 0.05.

### Animals

Animal experiments were permitted by the Animal Protection and Ethics Committee of the First Affiliated Hospital of Zhengzhou University. BALB/c nude mice (6-8 weeks) were purchased from Beijing Weitong Lihua Experimental Animal Technology Co., Ltd. (Beijing, China). For the experiment of Xenograft, MKN-45 cells (5 × 10^6^) were suspended in 200 μl normal saline and subcutaneously injected or through the tail vein. Tumor volume (mm^3^): V (Mm^3^) = S2 (Mm^2^) × L (Mm) / 2.

### Cell culture

Cell lines and stable-transfected cells with praja2 were purchased from CHI Scientific, Inc (Jiangsu, China). The cells were cultured with complete medium including 89% 1640 and 10% FBS, both were purchased from Biological Industries (Beit-Haemek, Israel) and maintained in incubator with 37° C and 5% of CO_2_ saturated humidity.

### Cell transfection and treatment

The MKN-45 cells were plated until the cell density reached 80% confluency of dishes to transfect. The plasmids transfected with Lipofectamine 2000 (Invitrogen, Carlsbad, CA, USA). 20 μM MG132 (IUP1011, Gene Operation) was added into cells for 6 h, 4 μM FGF2 (P5457, Abnova) was added into cells for 1 h. The plasmid of praja2 or KSR1 or small interfering RNA (si-RNA) of praja2 or KSR1 and were constructed by Genechem (Shanghai, China). The siRNA sequences of si-KSR1: 5′-UACCAUUAGAUUAGUUCUG-3′.

### qRT-PCR

RNA extraction was performed using trizol reagent. NanoDrop 8000 (Thermo Scientific, Waltham, MA, USA) was used to detect the concentration and purity of RNA. The single-stranded cDNAs were synthesized from 1 μg of RNA. The expression of mRNAs was quantified by RT-PCR with SYBR Green I (Thermo Fisher Scientific, Inc). Primer list: KSR1 (F: 5′-CTTTGCCTCTAGGGTCCG-3′, R: 5′-CGGACCCTAGAGGCAAAG-3′,), praja2 (F: 5′-AAGGCAAGAGGTGGATAA CAC-3′, R: 5′-AGG GCC ACT GCT ATC ACT T-3′), GADPH (F: 5′- CTCCTGCACCACCAACTGCT -3′, R: 5′- GGGCCATCCACAGTCTTCTG -3′).

### Immunohistochemical staining (IHC)

Paraffin sections of carcinoma were dewaxing to water in xylene and descending series of ethanol. We penetrated sections using 0.5% Triton X-100. After 3 times wash, we blocked sections with 50% goat serum. Then, sections were incubated with praja2 or KSR1 antibody overnight. We incubated the sections using secondary antibody followed by DAPI staining. The sections were photographed by light scope under an IX73 fluorescence microscope (Olympus, Valley, PA, USA) and analyzed by Image J software. Antibodies: KSR1 (4640S, Cell Signaling Technology), praja2 (abs141100, Absin).

### Western blot

After RIPA cleavage, we extracted total protein and measured with BCA method. After quantitative denaturation, protein electrophoresis membrane transfer and blocked. The first incubation and second incubation were carried out according to the operation steps. The expression of the protein was expressed by the gray value. Primary antibodies list: KSR1 (4640S, Cell Signaling Technology), praja2 (abs141100, Absin), p-MEK1/2 (Ser217/221) (41G9, Cell Signaling Technology), MEK1/2 (9122, Cell Signaling Technology), p-ERK1/2 (4370, Cell Signaling Technology), ERK1/2 (ab17942, Abcam), GADPH (ab181602, Abcam).

### CCK8 assay

MKN-45 cells were plated in 96-well plates and we used CCK8 assay to detect the cell viability. CCK8 (10 nmol/L; Beyotime Biotechnology, China) was added after curcumin treatment and incubated at 37° C. We measured the absorbance of 450 nm at 24, 48 and 72 h.

### Detection of apoptosis

The Annexin V-FITC/PI apoptosis kit was purchased from Solebao Company(Beijing, China), and an appropriate amount of logarithmic growth phase cells were washed twice with pre-cooled PBS. The MKN-45 cells were suspended with 500 ul of bound buffer, mixed with 5 ul of annexin V-FITC and PI, respectively, and placed at 25° C for 15 min. The apoptosis rate was determined by flow cytometry.

### Immunoprecipitation assay

The interaction of praja2 and KSR1 was detected using Co-immunoprecipitation kit (Pierce, USA) as previous described [16]. Briefly, HEK293 cells were transfected Flag-tagged praja2 or Flag-tagged mutant praja2, and isolating cell proteins after 48 h. Then, immunoblotting was used to detect Flag, KSR1 and Ub expression.

### EdU assay

Logarithmic growth phase cells were taken and seeded in 24-well plates with 1×10^5^ cells per well, and cultured to the normal growth stage. The cells were incubated with 200 μL diluted EdU solution (50 μM, Ribobio, Guangzhou RiboBio Co., Ltd., China) for 2 h. The cells were washed with PBS twice for 5 minutes each time. The nucleus was then labeled with DAPI and Edu positive cells were calculated by microscope.

### CHX assay

CHX assay was used to detect the role of praja2 on KSR1 stability. MKN-45 cells were transfected with si-praja2 or si-Scramble, then treated with 50 μg/mL cycloheximide (CHX, Sigma, C7698) for 0, 30, 60 and 90 min. And western blot was performed to determine the protein expression of praja2 and KSR1.

### Statistical analysis

Data were shown as mean±SD. Student’s t-test or one-way ANOVA was used to compare the groups. P<0.05 was considered significance.

## Supplementary Material

Supplementary Figures
