# Supplementary material for: Praja2 suppresses the growth of gastric cancer by ubiquitylation of KSR1 and inhibiting MEK-ERK signal pathways
Source: Aging (Albany NY). 2021 Jan 10;13(3):3886–97. doi: 10.18632/aging.202356 (PMC7906149; doi:10.18632/aging.202356)
Supplement: Supplementary Figures [file aging-13-202356-s001.pdf]

SUPPLEMENTARY FIGURES

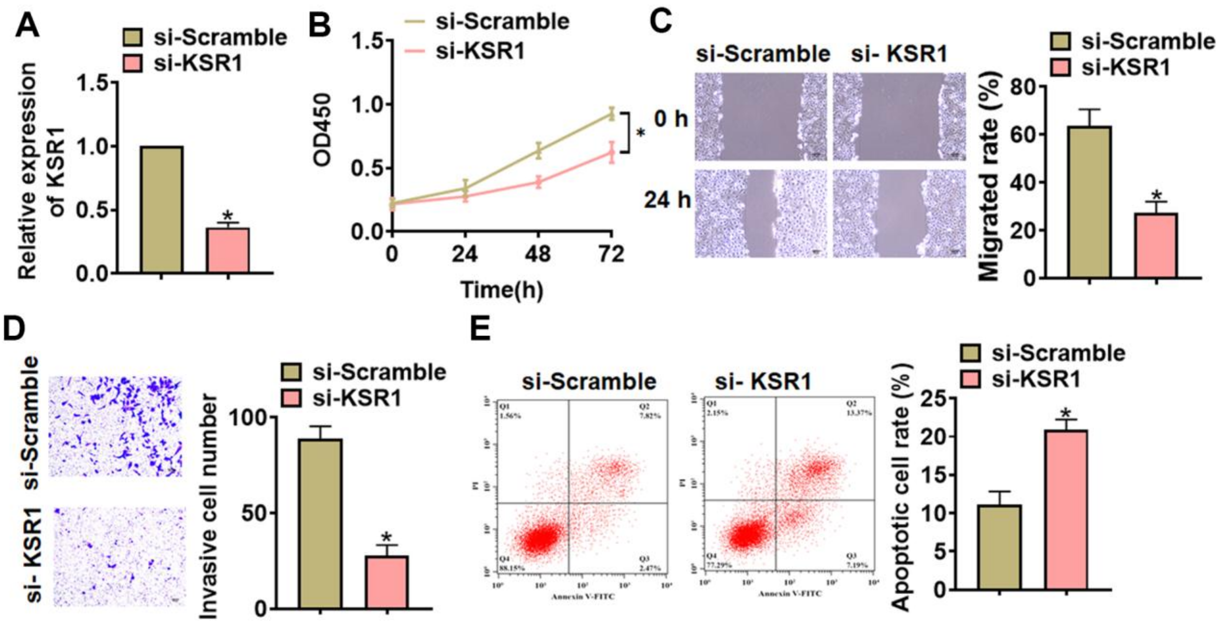

Supplementary Figure 1. Inhibition of KSR1 inhibited the migration and invasion of NCI-N87 cells. (**A**) The silencing efficiency of si-KSR1 in NCI-N87 cells. (**B**) CCK8 was to determine viability of NCI-N87 cells. (**C**) Wound healing assay was to evaluate migration of NCI-N87 cells. Scale bar, 60 µm. (**D**) Transwell assay was to examine invasion of NCI-N87 cells. Scale bar, 60 µm. (**E**) Flow cytometry assay was to determine apoptosis of NCI-N87 cells. Data are mean ± SD; *P < 0.05.

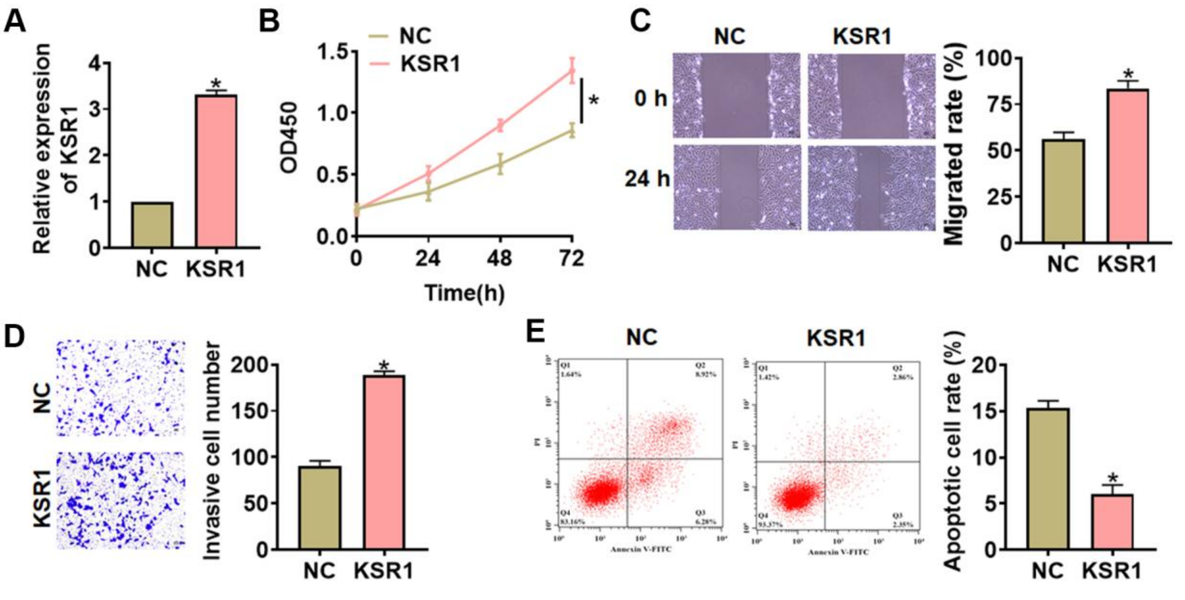

Supplementary Figure 2. KSR1 promoted the migration and invasion of NCI-N87 cells. (**A**) The transfection efficiency of KSR1 in NCI-N87 cells. (**B**) CCK8 for viability of NCI-N87 cells. (**C**) Wound healing assay for migration of NCI-N87 cells. Scale bar, 60 µm. (**D**) Transwell assay for invasion of NCI-N87 cells. Scale bar, 60 µm. (**E**) Flow cytometry assay for apoptosis of NCI-N87 cells. Data are mean ± SD; *P < 0.05.
